# Characterizing Breast Tumor Heterogeneity Through IVIM-DWI Parameters and Signal Decay Analysis

**DOI:** 10.3390/diagnostics15121499

**Published:** 2025-06-12

**Authors:** Si-Wa Chan, Chun-An Lin, Yen-Chieh Ouyang, Guan-Yuan Chen, Chein-I Chang, Chin-Yao Lin, Chih-Chiang Hung, Chih-Yean Lum, Kuo-Chung Wang, Ming-Cheng Liu

**Affiliations:** 1 Department of Medical Imaging, Taichung Veterans General Hospital, Taichung 407219, Taiwan; chan.siwa@gmail.com (S.-W.C.); penguin2046@gmail.com (M.-C.L.); 2 Department of Medical Imaging and Radiological Sciences, Central Taiwan University of Science and Technology, Taichung 40601, Taiwan; 3 Department of Post-Baccalaureate Medicine, College of Medicine, National Chung Hsing University, Taichung 402202, Taiwan; 4 Department of Electrical Engineering, National Chung Hsing University, Taichung 402202, Taiwan; julian135707@gmail.com; 5 Department of Communication Engineering, National Chung Hsing University, Taichung 402202, Taiwan; 0551109@nkust.edu.tw; 6 Remote Sensing Signal and Image Processing Laboratory, University of Maryland, College Park, MD 20742, USA; cchang@umbc.edu; 7 Department of Electrical Engineering, National Cheng Kung University, Tainan City 70142, Taiwan; 8 Department of Breast Medical Center, Taichung Tzu Chi Hospital, Taichung 427213, Taiwan; chinyao1014@gmail.com; 9 Division of Breast Surgery, Department of Surgery, Taichung Veterans General Hospital, Taichung 407219, Taiwan; mental7330@gmail.com (C.-C.H.); memorygiann@gmail.com (C.-Y.L.); kcwang@vghtc.gov.tw (K.-C.W.); 10 School of Medicine, College of Medicine, National Yang Ming Chiao Tung University, Taipei 112304, Taiwan

**Keywords:** breast cancer, diffusion-weighted imaging (DWI), intravoxel incoherent motion (IVIM), dynamic contrast-enhanced MRI (DCE-MRI), deep neural networks (DNNs), apparent diffusion coefficient (ADC)

## Abstract

**Background/Objectives:** This research presents a novel analytical method for breast tumor characterization and tissue classification by leveraging intravoxel incoherent motion diffusion-weighted imaging (IVIM-DWI) combined with hyperspectral imaging techniques and deep learning. Traditionally, dynamic contrast-enhanced MRI (DCE-MRI) is employed for breast tumor diagnosis, but it involves gadolinium-based contrast agents, which carry potential health risks. IVIM imaging extends conventional diffusion-weighted imaging (DWI) by explicitly separating the signal decay into components representing true molecular diffusion (D) and microcirculation of capillary blood (pseudo-diffusion or D*). This separation allows for a more comprehensive, non-invasive assessment of tissue characteristics without the need for contrast agents, thereby offering a safer alternative for breast cancer diagnosis. The primary purpose of this study was to evaluate different methods for breast tumor characterization using IVIM-DWI data treated as hyperspectral image stacks. Dice similarity coefficients and Jaccard indices were specifically used to evaluate the spatial segmentation accuracy of tumor boundaries, confirmed by experienced physicians on dynamic contrast-enhanced MRI (DCE-MRI), emphasizing detailed tumor characterization rather than binary diagnosis of cancer. **Methods:** The data source for this study consisted of breast MRI scans obtained from 22 patients diagnosed with mass-type breast cancer, resulting in 22 distinct mass tumor cases analyzed. MR images were acquired using a 3T MRI system (Discovery MR750 3.0 Tesla, GE Healthcare, Chicago, IL, USA) with axial IVIM sequences and a bipolar pulsed gradient spin echo sequence. Multiple b-values ranging from 0 to 2500 s/mm^2^ were utilized, specifically thirteen original b-values (0, 15, 30, 45, 60, 100, 200, 400, 600, 1000, 1500, 2000, and 2500 s/mm^2^), with the last four b-value images replicated once for a total of 17 bands used in the analysis. The methodology involved several steps: acquisition of multi-b-value IVIM-DWI images, image pre-processing, including correction for motion and intensity inhomogeneity, treating the multi-b-value data as hyperspectral image stacks, applying hyperspectral techniques like band expansion, and evaluating three tumor detection methods: kernel-based constrained energy minimization (KCEM), iterative KCEM (I-KCEM), and deep neural networks (DNNs). The comparisons were assessed by evaluating the similarity of the detection results from each method to ground truth tumor areas, which were manually drawn on DCE-MRI images and confirmed by experienced physicians. Similarity was quantitatively measured using the Dice similarity coefficient and the Jaccard index. Additionally, the performance of the detectors was evaluated using 3D-ROC analysis and its derived criteria (*AUC_OD_*, *AUC_TD_*, *AUC_BS_*, *AUC_TD_*_−*BS*_, *AUC_ODP_*, *AUC_SNPR_*). **Results:** The findings objectively demonstrated that the DNN method achieved superior performance in breast tumor detection compared to KCEM and I-KCEM. Specifically, the DNN yielded a Dice similarity coefficient of 86.56% and a Jaccard index of 76.30%, whereas KCEM achieved 78.49% (Dice) and 64.60% (Jaccard), and I-KCEM achieved 78.55% (Dice) and 61.37% (Jaccard). Evaluation using 3D-ROC analysis also indicated that the DNN was the best detector based on metrics like target detection rate and overall effectiveness. The DNN model further exhibited the capability to identify tumor heterogeneity, differentiating high- and low-cellularity regions. Quantitative parameters, including apparent diffusion coefficient (ADC), pure diffusion coefficient (D), pseudo-diffusion coefficient (D*), and perfusion fraction (PF), were calculated and analyzed, providing insights into the diffusion characteristics of different breast tissues. Analysis of signal intensity decay curves generated from these parameters further illustrated distinct diffusion patterns and confirmed that high cellularity tumor regions showed greater water molecule confinement compared to low cellularity regions. **Conclusions:** This study highlights the potential of combining IVIM-DWI, hyperspectral imaging techniques, and deep learning as a robust, safe, and effective non-invasive diagnostic tool for breast cancer, offering a valuable alternative to contrast-enhanced methods by providing detailed information about tissue microstructure and heterogeneity without the need for contrast agents.

## 1. Introduction

Breast cancer is a leading cause of cancer-related mortality, with approximately 2.3 million new cases diagnosed globally in 2020 [1]. Early detection of breast cancer significantly improves survival rates, driving the adoption of advanced imaging techniques, such as magnetic resonance imaging (MRI), for screening and diagnosis. Unlike mammography, which poses radiation risks, MRI offers superior sensitivity in detecting breast tumors, especially in patients with dense breast tissue [2].

Dynamic contrast-enhanced MRI (DCE-MRI) is often used for breast cancer detection. However, DCE-MRI involves the use of gadolinium-based contrast agents, which carry potential risks, such as nephrogenic systemic fibrosis and gadolinium retention in the body, particularly for patients with renal insufficiency [3]. Moreover, distinguishing between benign and malignant tumors using DCE-MRI can be challenging, as the enhancement patterns may overlap. To address these limitations, diffusion-weighted imaging (DWI) and intravoxel incoherent motion (IVIM) imaging have emerged as promising alternatives for non-invasive breast cancer detection without the use of contrast agents.

DWI is an MRI technique that measures the random Brownian motion of water molecules within tissues, providing insights into tissue microstructure. Regions with restricted diffusion, often indicative of high cellularity, are characteristic of malignant tumors [4]. IVIM imaging extends the capabilities of DWI by separating true molecular diffusion from the microcirculation of blood in the capillaries, allowing for a more comprehensive assessment of tissue characteristics [5,6]. Quantitative parameters such as the apparent diffusion coefficient (ADC), pure diffusion coefficient (D), pseudo-diffusion coefficient (D*), and perfusion fraction (PF) can be derived from IVIM, providing valuable information for differentiating between malignant and non-malignant tissues.

Several studies have demonstrated the effectiveness of IVIM-DWI in diagnosing malignancies in various organs, including the liver and prostate, with these quantitative parameters being accepted in clinical settings [7,8,9]. In this study, we extend the application of IVIM-DWI to breast cancer detection, incorporating hyperspectral imaging techniques to enhance the analysis of diffusion-weighted images at multiple b-values. Hyperspectral imaging allows for the extraction of detailed spectral information from each voxel, improving the ability to distinguish between different tissue types [10].

IVIM imaging explicitly separates the signal decay into components representing pure molecular diffusion (D) and capillary microcirculation (pseudo-diffusion or D*). By analyzing these distinct diffusion components, our method provides a comprehensive and non-invasive assessment of tissue characteristics without the need for contrast agents, thus offering safer clinical implications.

To achieve precise characterization of breast tumors, we utilize kernel-based constrained energy minimization (KCEM) and iterative KCEM methods for initial tumor region detection. These methods are effective in identifying spectral signatures associated with malignant tissues. Additionally, we employ deep neural networks (DNNs) to perform detailed spatial segmentation and characterization of breast tumor tissues based on the extracted features. DNNs have shown great potential in medical imaging due to their ability to learn complex patterns and features from large datasets [11]. By combining traditional hyperspectral imaging methods with modern deep learning techniques, we aim to develop a robust framework for non-invasive breast cancer detection.

The objectives of this study were (1) to characterize breast tumor heterogeneity using IVIM-DWI combined with hyperspectral imaging; (2) to compare three tumor detection methods—KCEM, iterative KCEM (I-KCEM), and deep neural networks (DNNs)—based on their ability to accurately delineate tumor boundaries; and (3) to assess the effectiveness of quantitative IVIM parameters in distinguishing different breast tissue types.

### 1.1. Magnetic Resonance Imaging

The current medical imaging technique known as magnetic resonance imaging (MRI) utilizes radio frequency pulses and magnetic fields to examine the energy within various tissues [11,12,13,14]. By harnessing the hydrogen atoms present in water molecules, which are abundant in the human body, MRI captures emitted signals and reconstructs them into images. A significant advantage of MRI is its non-invasive nature, as it does not involve radiation exposure, making it a safe option for clinical use [15]. MRI offers high-resolution anatomical images of different body parts. Its capability to visualize soft tissues with remarkable clarity has established it as an indispensable tool in medical diagnosis.

### 1.2. Diffusion-Weighted Imaging

Diffusion-weighted imaging (DWI) captures the diffusion motion of water molecules, highlighting areas with restricted diffusion associated with high cell density, such as tumors [16,17,18,19]. The apparent diffusion coefficient (ADC), derived from DWI, quantifies diffusion extent and has proven valuable for distinguishing benign and malignant tissues. However, the ADC calculation based on a mono-exponential model has limitations, prompting the use of more advanced techniques like intravoxel incoherent motion (IVIM).

### 1.3. Intravoxel Incoherent Motion

The intravoxel incoherent motion (IVIM) imaging technique [20] enhances traditional diffusion-weighted imaging by distinguishing between two distinct types of water molecule movements within biological tissues: true molecular diffusion (D) and microvascular perfusion, termed pseudo-diffusion (D*) [21,22,23]. IVIM also quantifies the perfusion fraction (PF), representing the proportion of signal arising from blood microcirculation. These parameters—D, D*, and PF—are particularly valuable for capturing detailed tissue characteristics, such as microvascular density and cellularity, enabling effective differentiation between malignant and benign tissues based on their unique diffusion and perfusion profiles.

### 1.4. Apparent Diffusion Coefficient

The apparent diffusion coefficient (ADC) is a critical parameter derived from diffusion-weighted imaging (DWI) that quantifies water molecule diffusion within tissues. Typically, higher ADC values correspond to greater molecular mobility and indicate less restrictive environments, whereas lower ADC values often reflect dense cellularity or restricted diffusion environments [20,21,22,24]. However, conventional ADC measurements rely on a simplified mono-exponential diffusion model, assuming a Gaussian diffusion process that may not fully represent the complexity of actual biological diffusion phenomena. To obtain a more accurate representation of diffusion processes, higher b-values (usually greater than 200 $s/{\mathrm{mm}}^{2}$) are often utilized, providing better characterization of tissue microstructure and improving the distinction between benign and malignant lesions.

### 1.5. Band Expansion Process

The band expansion process (BEP), originally proposed by Ren and Chang, addresses the inherent limitation of conventional MR imaging, which typically involves acquiring a limited number of images. Standard MRI sequences may not fully capture the rich spectral information essential for detailed tissue characterization. The BEP generates additional image bands through the nonlinear correlation between original MR images obtained at different b-values. This nonlinear generation of supplementary bands significantly enhances the hyperspectral characteristics within MR data, resulting in improved tissue discrimination capability and higher classification accuracy [25].

### 1.6. Deep Neural Networks

Deep neural networks (DNNs) have gained popularity in medical image analysis due to their superior ability to automatically learn and recognize complex patterns within data [26]. Unlike traditional analysis methods, DNNs effectively perform hierarchical feature extraction, enabling accurate classification and differentiation of subtle image characteristics. The adaptability and effectiveness of DNNs make them particularly valuable for tasks involving sophisticated image interpretation, such as tumor detection and tissue characterization.

### 1.7. Advancements in Medical Imaging and Artificial Intelligence in Cancer

Recent studies integrating MRI with artificial intelligence (AI) have significantly advanced cancer diagnosis. AI-driven approaches utilizing DWI and IVIM have effectively characterized breast, prostate, and pancreatic cancers, highlighting their potential clinical utility [27,28,29,30,31,32,33].

Previous studies have utilized whole-lesion histogram analysis based on diffusion-weighted imaging for breast lesion characterization, employing advanced diffusion models such as the stretched-exponential diffusion model [34]. Our study extends this foundation by integrating hyperspectral imaging and deep learning techniques, which enable more detailed quantitative and spatial characterization of breast tumor heterogeneity. Additionally, previous breast tissue classification methods based on high-resolution MR imaging faced limitations due to pixel misalignment when mapping classification results onto IVIM images. To overcome this limitation, our study directly employs IVIM images, aiming for more precise and reliable tumor quantification.

## 2. Materials and Methods

**Figure 1:**
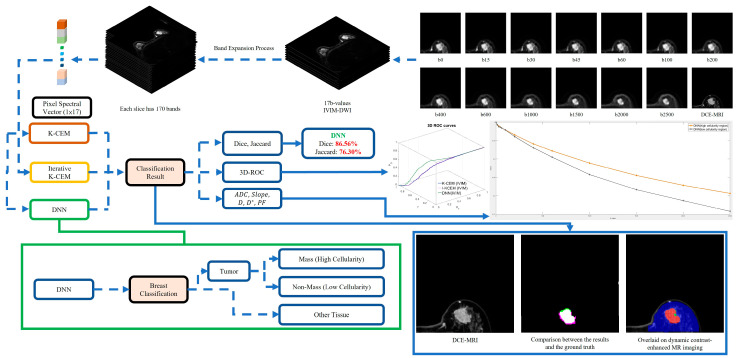
Flowchart of image pre-processing, band expansion from 17 to 170 hyperspectral bands, tumor detection methods (KCEM, I-KCEM, DNN), quantitative evaluation using Dice similarity coefficients, Jaccard indices, and 3D-ROC analysis, and the generation of signal intensity decay curves to characterize tumor heterogeneity.

Figure 1 presents a comprehensive hyperspectral image analysis flowchart designed to characterize breast tumor heterogeneity using IVIM-DWI techniques. 

The methodology comprises the following four interconnected stages:

(i) Image pre-processing, involving intensity normalization, pixel alignment, and breast masking to enhance MRI image clarity and consistency (upper-right section in Figure 1);

(ii) Band expansion process, initially utilizing 17 diffusion-weighted images corresponding to multiple b-values (ranging from 0 to 2500 $s/{\mathrm{mm}}^{2}$). These images were expanded into 170 hyperspectral bands through nonlinear correlation methods, enhancing spectral representation for improved tissue characterization (upper-central section in Figure 1);

(iii) Breast tumor detection, employing kernel-based constrained energy minimization (KCEM), iterative KCEM (I-KCEM), and deep neural networks (DNNs). These methods identify tumor regions based on spectral features from the expanded hyperspectral bands. Segmentation performance was quantitatively evaluated using the Dice similarity coefficient and Jaccard index. Importantly, these metrics were not utilized to assess binary diagnostic accuracy (presence or absence of cancer). Instead, they were specifically chosen to quantitatively evaluate the spatial accuracy of the tumor segmentation methods within the IVIM-DWI images. The segmentation results from each computational method (KCEM, I-KCEM, and DNN) were compared to manually delineated tumor boundaries defined by experienced radiologists on dynamic contrast-enhanced MRI (DCE-MRI), serving as the ground truth for evaluating the accuracy of tumor region delineation. Additionally, the performance of each detection method was validated using 3D receiver operating characteristic (3D-ROC) analysis (middle to lower section in Figure 1);

(iv) Quantitative analysis, calculating key diffusion parameters—apparent diffusion coefficient (ADC), pure diffusion coefficient (D), pseudo-diffusion coefficient (D*), and perfusion fraction (PF)—using mono-exponential and bi-exponential models [24]. These parameters were further used to generate signal intensity decay curves, clearly distinguishing regions of high and low cellularity within breast tissues (right-middle section in Figure 1).

This integrated workflow leverages advanced hyperspectral imaging and machine learning techniques to precisely differentiate between tumor and normal breast tissues, enhancing the capabilities for non-invasive breast cancer detection.

### 2.1. Materials

In this study, MR images were obtained using a 3T MRI scanner (Discovery MR750 3.0 Tesla, GE Healthcare, Chicago, IL, USA). Our dataset consisted of breast MRI scans from 22 female patients diagnosed with mass-type breast tumors. All cases were retrospectively collected from Taichung Veterans General Hospital, Taiwan, and were confirmed histologically by experienced breast radiologists and surgeons.

For breast MRI, patients lie face-down, placing their breasts into a specialized coil designed to capture clear images. The MRI scanner then generated detailed images by detecting signals naturally emitted from hydrogen atoms within body tissues, responding to controlled magnetic fields and radio waves. To capture images sensitive to tissue diffusion characteristics, diffusion-weighted images (DWIs) were obtained using a specialized MRI sequence (a bipolar pulsed gradient spin echo sequence, PGSE), with multiple levels of diffusion sensitivity known as b-values ranging from 0 to 2500 $s/{\mathrm{mm}}^{2}$.

Axial IVIM images were acquired using echo planar imaging (EPI) single spin echoes, covering both breasts (repetition time (TR)/echo time (TE): 3000/74 ms, reconstructed voxel size: 3 × 3 × 4 ${\mathrm{mm}}^{3}$, field of view (FOV): 320 × 320 mm^2^, matrix: 256 × 256 × 12). Spectral pre-saturation inversion recovery and diffusion sensitization in the anterior-posterior direction were performed with weighting factors (b) of 0, 15, 30, 45, 60, 100, 200, 400, 600, 1000, 1500, 2000, and 2500 $s/{\mathrm{mm}}^{2}$.

### 2.2. Image Pre-Processing

Some errors existed in the obtained case images, e.g., the images shifted because of the patient’s breathing behavior. We corrected the images for N3 (non-parametric, non-uniform intensity, normalization) and used Avizo software (version 7.0, Thermo Fisher Scientific, Waltham, MA, USA) to match the pixels of the images to obtain clearer MR images. The corrected images were generated using a semi-automatic method to create breast masks, where the masks were the breast areas of interest, as follows:

Step 1: Rectify the image using the N3 model. This correction method improves image intensity inhomogeneity independent of the pulse sequence.

Step 2: MR image acquisition is a time-consuming process, and the patient’s breathing can cause small changes in the image during MR imaging. Therefore, the number of images may vary when using different MR methods and parameters. The Avizo software camera helps match all pixels on the slice for a clearer MR image.

**Figure 2:**
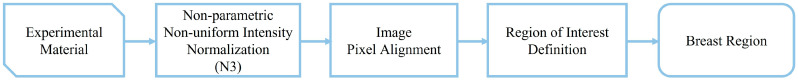
Flowchart of image pre-processing.

Step 3: A semi-automatic method is used to generate the breast masks. The mask segments the breast area and removes the nipples, muscle, skin, bone, and other regions to obtain the breast area of interest for experimentation, as illustrated in Figure 2.

### 2.3. Hyperspectral Techniques

A multispectral image, where each spectral band image can be viewed as an image acquired by a specific pulse sequence, has been commonly used for research [11]. Hyperspectral imaging improves on the traditional multispectral imaging method by providing superior spectral resolution and hundreds of continuous spectral bands instead of the dozens of spectral bands used in multispectral imaging. Thus, hyperspectral imaging can be easily applied to brains that are more frequently used in MRI [12,13,14].

MRI has revealed several different breast-tissue characteristics with better quality than traditional techniques. Furthermore, different pulse sequences can be used in MRI and radiography to enhance the contrast in different tissues. Therefore, we distinguished similar structures from soft tissues, which increased the number of tissue features and lesions. One of the aims of this study is to present diagnostic results using different MR processing techniques.

### 2.4. IVIM Parameter Calculation

Intravoxel incoherent motion (IVIM) imaging was employed to acquire diffusion-weighted (DW) images using multiple diffusion-weighting factors (b-values). This technique separates diffusion from perfusion effects within tissues by utilizing a bi-exponential signal decay model. Specifically, the bi-exponential IVIM model is expressed as follows:

(1) $$S_{bi}=S_{b0}*\left[\left(1-PF\right)*\exp\left(-b\cdot D\right)/+PF*\exp\left(-b\cdot D*\right)\right]$$

In this equation, $S_{bi}$ and $S_{b0}$ represent signal intensities at specific b-values and the baseline (b = 0 $s/{\mathrm{mm}}^{2}$), respectively. Parameter D is the true diffusion coefficient, reflecting molecular diffusion, while D* (pseudo-diffusion coefficient) relates to perfusion effects from microvascular blood flow. The perfusion fraction (PF) quantifies the fractional contribution of microvascular perfusion within the voxel.

At low b-values (<100 $s/{\mathrm{mm}}^{2}$), the IVIM signal predominantly reflects microvascular perfusion, whereas at higher b-values (>100 $s/{\mathrm{mm}}^{2}$), diffusion becomes the dominant factor. To independently calculate the pure diffusion coefficient (D), the simplified mono-exponential formula was applied at high b-values as follows:

(2) $$S_{bi}=S_{b0}\exp\left(-bi\cdot D\right)$$

Similarly, D* was estimated using the low b-values region (<100 $s/{\mathrm{mm}}^{2}$). The perfusion fraction (PF) was computed according to the following equation:

(3) $$PF=\frac{\left(S_{bi}-S_{b0}\right)}{S_{b0}}$$

**Figure 3:**
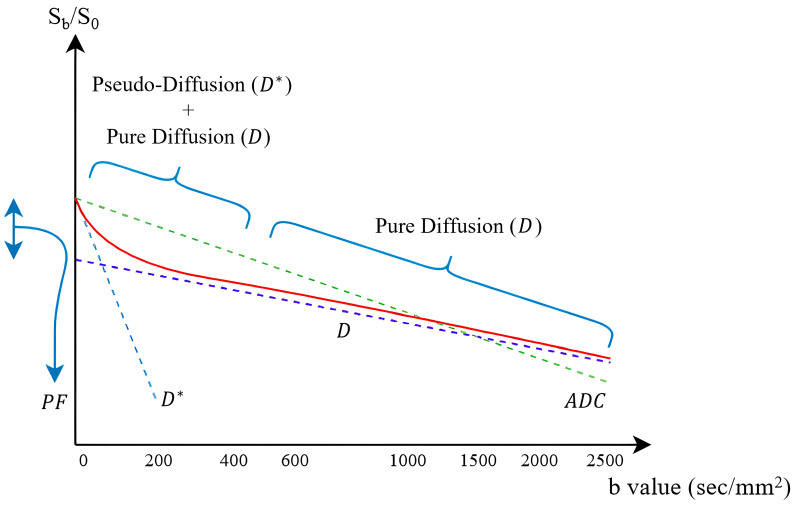
IVIM signal decay curves. The red solid line represents the measured IVIM signal, the green dotted line represents the ADC component, the purple dotted line indicates the pure diffusion coefficient (D), and the blue dotted line illustrates the pseudo-diffusion coefficient (D*).

The calculated signal intensities at various b-values were plotted to form IVIM signal intensity decay curves. Analysis of these curves allows differentiation among tissues based on distinct diffusion and perfusion characteristics, as illustrated in Figure 3.

### 2.5. Apparent Diffusion Coefficient Calculation

The apparent diffusion coefficient (ADC) was calculated to quantify the diffusion extent of water molecules within tissues. The ADC measurement relies on diffusion-weighted imaging (DWI) using multiple diffusion-weighting factors known as b-values. The b-value indicates diffusion sensitivity, determined by the strength, duration, and timing of the diffusion gradients applied during imaging, expressed mathematically as follows:

(4) $$b=\gamma^{2}g^{2}\delta^{2}\left(\Delta -\frac{\delta }{3}\right)$$

where b is the diffusion sensitivity factor, γ is the proton gyromagnetic ratio, g is the gradient strength, δ is the gradient duration, and Δ is the time between gradient pulses.

The ADC was computed using a mono-exponential model, comparing the signal intensities at two selected b-values according to the following equation:

(5) $$ADC=\frac{\ln\left(\frac{S_{bi}}{S_{b0}}\right)}{b_{b0}-b_{bi}}$$

In this formula, $S_{b0}$ and $S_{bi}$ represent signal intensities measured at the baseline (b = 0 $s/{\mathrm{mm}}^{2}$) and at a higher b-value (bi), respectively. Generally, an increase in the ADC reflects greater diffusion of water molecules, indicating less restrictive tissues.

### 2.6. Breast Tumor Detection

Hyperspectral techniques are used for breast tumor detection. This experiment used the IVIM-DW image cube pixels as the input. We imagined a set of spectral vectors for the breast tissue because IVIM-DW images have 13 b-values, and each of the last four b-value images is replicated once to improve tumor detection accuracy. We used KCEM, I-KCEM, and DNNs to detect tumor locations on the IVIM-DW images to classify mass tumors into high- and low-cellularity locations.

Before using the tumor detection method, we employed some common processing methods for hyperspectral images, such as the expansion and selection of the spectral band and the definition of the target spectrum. These methods include the band expansion process (BEP) [14,25], automatic target generation process (ATGP) [35], and spectral angle mapper (SAM) [36,37,38], and they are introduced in this given order. Hyperspectral images contain hundreds of bands that are used for detection. The DW imaging has 13 b-values that can be considered as 13 bands; however, the higher the b-value, the clearer the tumor signal. We considered the last four bands again for a total of 17 bands. However, these 17 bands contained relatively less data than those in the hyperspectral images. The BEP nonlinear method is used to increase the useful data by randomly selecting two oriented DW images. By correlating two DW images with different b-values, we generate a new image, which is a set of second-order statistical bands [14]. The BEP method uses the cross-correlation and autocorrelation of all original images to create more second-order statistical bands. ATGP is an unsupervised classification method used in hyperspectral orthogonal subspace projection (OSP) classifiers [35]. When we detect the unknown formation, ATGP automatically performs target detection and classification without prior probability testing, and it is often used in anomaly detection where breast tumors are part of the anomaly. SAM is an automated method that can be used to directly compare image spectra to known spectra or endmembers as vectors and to calculate the spectral angle between them. Before employing SAM for spectrum classification, we must supply a set of known target spectra for comparison with the image spectrum. The target spectrum identifies a similar vector within the image spectrum, judging based on the angle θ between them. A smaller angle indicates a higher similarity between the image and target spectra, whereas a larger angle suggests a lower similarity. The SAM is expressed as follows:

(6) $$\theta =cos^{-1}\frac{\sum XY}{\sqrt{\sum {\left(X\right)}^{2}{\left(Y\right)}^{2}}}$$

where θ, X, and Y represent the angle between the target and image spectra, the vector of the target spectrum, and the vector of the image spectrum, respectively. When θ is equal to 90, the target spectrum is the same as the image spectrum.

**Figure 4:**
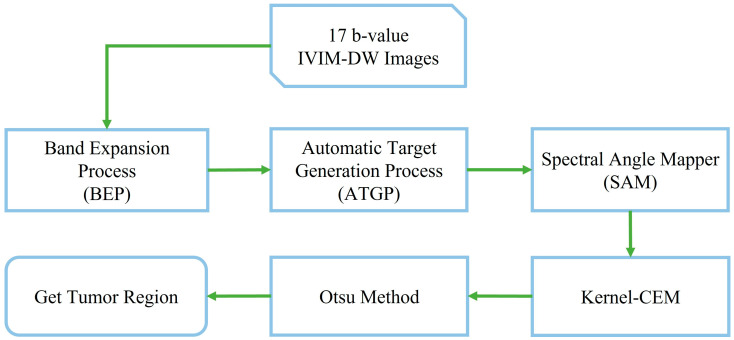
Flowchart of tumor detection using K-CEM.

Figure 4 shows the workflow using the K-CEM method, which was first processed using the hyperspectral method described above and then analogously used to detect breast tumors on IVIM-DW images. Constrained energy minimization (CEM) is widely used for target detection in hyperspectral remote sensing imagery. This method detects the target signal using constraints and minimizes the average energy of the output by suppressing noise and unknown sources [39]. However, the CEM detection method requires the signature of the target to be known before it can be detected.

The CEM is a linear filter, and therefore, the nonlinear separability in dealing with non-linearly generated DW imaging presents a problem. Thus, extending the CEM to a kernel version, called K-CEM, may be more effective for object detection [40,41]. The iterative K-CEM method addresses the potential problem of the initial training samples not being suitable. However, we conducted an initial training sample search.

The SAM method may not be sufficiently accurate. Therefore, we used 10% of the K-CEM results as the training sample for the new K-CEM, calculated the difference rate between the old and new K-CEM results, and terminated the experiment when the difference was less than 1%.

**Figure 5:**
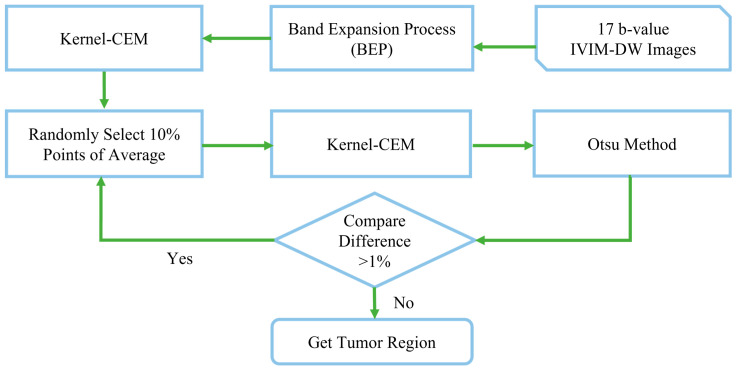
Flowchart of tumor detection using iterative KCEM (I-KCEM).

This process is called iterative K-CEM, and its flowchart is shown in Figure 5.

### 2.7. Tumor Detection Using a Deep Neural Network (DNN)

**Figure 6:**
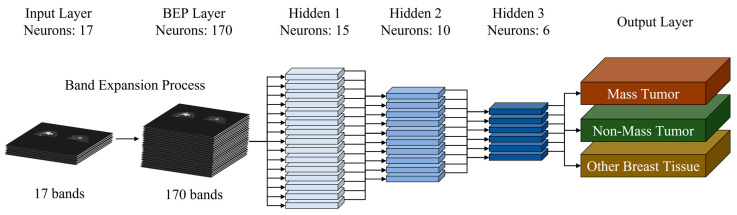
Deep neural network architecture for tumor detection.

Deep neural networks (DNNs) have been widely researched in recent years and have shown good results in numerical analyses, object segmentation, and object recognition [26]. In convolutional neural networks (CNNs), the input image is used to find features using the convolutional layer, and then, the features are filtered by a pooling layer to achieve classification. However, this method is often used to recognize the shape of an object and to classify the input image by finding the edges. The shape of breast tumors is not fixed, and therefore, this method is not suitable. We use a fully connected DNN to classify the hyperspectral vectors of single-point pixels in the breast; the architecture is presented in Figure 6.

The input to the neural network is derived directly from the diffusion-weighted images (DWIs) measured at different diffusion weightings (b-values) for each voxel. Specifically, the voxel-wise signal intensities at multiple b-values are collected and preprocessed through background suppression and normalization using min-max scaling. The study used 17 b-values (b = 2500, 2500, 2000, 2000, 1500, 1500, 1000, 1000, 600, 400, 200, 100, 60, 45, 30, 15, and 0 $s/{\mathrm{mm}}^{2}$) for the DW-MRI acquisition. Therefore, the input layer of the DNN comprises neurons equal to the number of these b-values, each receiving the normalized signal intensity at a specific b-value for a given voxel. In essence, for each voxel in the DW-MRI data, the neural network receives a series of normalized signal intensities corresponding to the different diffusion weightings applied during the acquisition.

The network then processes this input to estimate the IVIM parameters (ADC, D, D*, and PF) for that voxel.

The accuracy of K-CEM for hyperspectral breast tumor detection is approximately 70%; however, we improved the detection accuracy by transferring cases with good detection results to the DNN for learning. For training the neural networks, we optimized the values using the Levenberg–Marquardt (LM) method [42]. The LM method improves the iterative algorithm and combines the advantages of the gradient descent method with the Gaussian–Newton method [43], which is used to solve nonlinear least-squares problems depending on the number of iterations.

The algorithm can be expressed as follows:

(7) $$x_{k+1}=x_{k}-{\left(J_{k}^{T}J_{k}+\mu I\right)}^{-1}J_{k}e_{k}$$

where x, e, and J represent the weight vector, training error, and Jacobian matrix, respectively. During the training process, the parameter changes with the iteration *µ*, and we use the Gauss–Newton algorithm or the gradient descent algorithm based on the algorithm used to approximate the results of the Gauss–Newton algorithm. This LM algorithm can be expressed as follows:

(8) $$x_{k+1}=x_{k}-{\left(J_{k}^{T}J_{k}\right)}^{-1}J_{k}e_{k}$$

To avoid oscillation of the search for the optimal value direction during later stages of the calculation, µ approaches ∞, and the LM algorithm approximates the gradient descent method. This algorithm can be expressed as follows:

(9) $$x_{k+1}=x_{k}-\alpha g_{k}$$

(10) $$g={\left[\frac{\partial E}{\partial x_{1}}\;\frac{\partial E}{\partial x_{2}}\;\ldots \;\frac{\partial E}{\partial x_{N}}\right]}^{T}$$

where α is the learning rate (step size), and g is the gradient.

We use the LM algorithm to approximate the Gaussian–Newton method as soon as possible. This method is fast and accurate for determining the error near the minimum value.

## 3. Results

All experiments and the proposed methods were performed using MATLAB 2021. The PC specifications are as follows: Intel® Core™ i9-9900K processor (3.60 GHz, Intel Corporation, Santa Clara, CA, USA), 32 GB RAM, and an NVIDIA GeForce RTX 2080 GPU (NVIDIA Corporation, Santa Clara, CA, USA).

In this section, we first present a comparison of the spatial accuracy among the three tumor detection methods—kernel-based constrained energy minimization (KCEM), iterative KCEM (I-KCEM), and deep neural networks (DNNs). Subsequently, we evaluate the quantitative IVIM parameters (ADC, D, D*, and PF) that distinguish tumor heterogeneity. Finally, we summarize the overall performance metrics and provide specific case examples to clearly illustrate our findings.

### 3.1. Datasets

Our dataset consisted of breast MRI scans obtained from 22 patients diagnosed with breast cancer, each presenting a mass-type tumor. In total, 22 distinct mass tumor cases were studied and analyzed.

**Figure 7:**
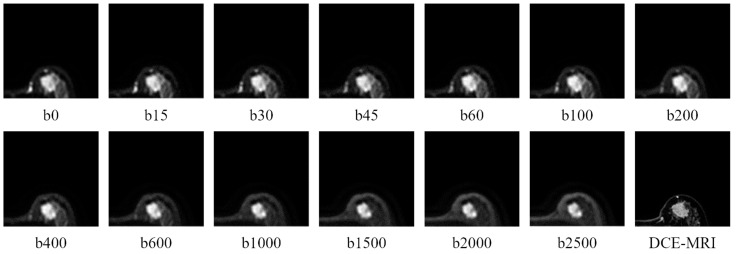
IVIM-DW images of the left breast with a tumor at different b-values (0~2500) and DCE-MR imaging of the left breast.

Figure 7 shows one case of IVIM-DW images with a tumor and the dynamic contrast-enhanced (DCE) MRIs of mass units with different b values. All the DCE-MR images clearly indicated the tumor location. We drew part of the tumor in the DCE-MRI images, and all the cases were confirmed by experienced physicians and used as our ground truth.

### 3.2. Similarity Evaluation Criteria

**Figure 8:**
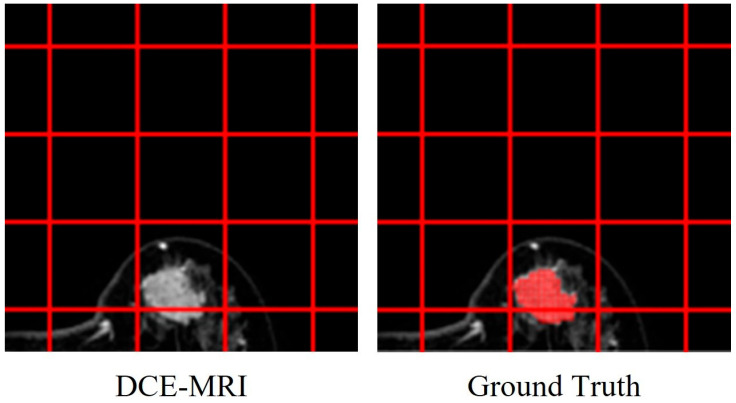
DCE-MR image with a tumor and the tumor ground truth area drawn on the DCE-MR image.

We normalized the segmentation results across all cases. The Dice [44] similarity coefficient and Jaccard [45] index were utilized specifically to evaluate the accuracy of tumor segmentation by comparing IVIM-based segmentation results to manually delineated tumor boundaries from dynamic contrast-enhanced MRI images. Figure 8 illustrates an example of the manually delineated tumor boundaries (ground truth) on a DCE-MR image used as a reference for these comparisons. It should be clarified that the Dice similarity coefficient and Jaccard index reported here reflect the spatial agreement between computationally segmented tumor regions and the expert-defined tumor boundaries. Specifically, the Dice coefficient of 86.56% achieved by the DNN model indicates high overlap and spatial accuracy compared with the tumor regions delineated by experienced radiologists rather than evaluating the presence or absence of cancer itself.

The Dice coefficient was primarily used to evaluate the similarity between the segmentation results and the ground truth. The results indicate that the closer the Dice coefficient is to 1, the more similar the segmentation results. The Jaccard index evaluates whether two segmented regions share a common area and calculates the difference between the two. The closer the Jaccard index is to 1, the higher the similarity between the segmented regions.

**Figure 9:**
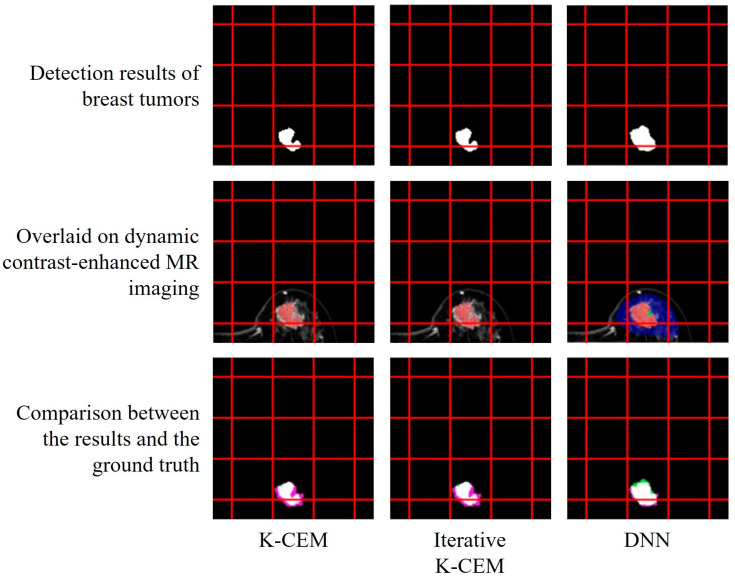
Results and similarity comparison of different detectors for breast tumor detection.

Figure 9 shows the similarity comparison results for the segmentation methods. Table 1 lists the similarity coefficients between the segmentation results of the various methods and the ground truth. The Dice and Jaccard equations are, respectively, given as follows:

(11) $$Dice\left(A,B\right)=\frac{2\left|A\cap B\right|}{\left|A\right|+\left|B\right|}$$

(12) $$Jaccard\left(A,B\right)=\frac{\left|A\cap B\right|}{\left|A\cup B\right|}$$

where A and B represent the images that we want to compare. The mammary gland detection results of the three detectors are shown in Figure 9, and the results are mapped to DCE-MR imaging. Figure 9 shows a comparison between the detection results and the ground truth. The white and red areas represent the ground truth ranges, and the white and green areas represent the detection results of the detector.

### 3.3. Evaluation Criteria of the Detector

**Figure 10:**
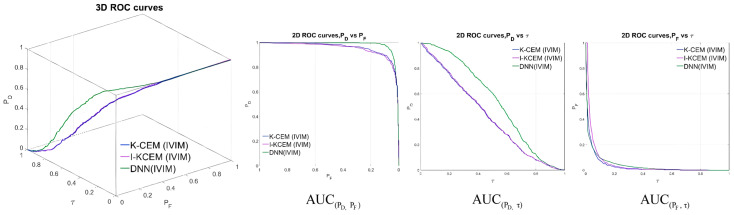
3D-ROC curves and 2D-ROC curves of all detectors.

We introduce 3D-ROC analysis [46], which is commonly used for signal detection, to evaluate the detector performance [47,48,49,50,51,52]. Three 2D-ROC curves for the detector, namely (P_D_, P_F_), (P_D_, τ), and (P_F_, τ), can be generated from the 3D-ROC curve, as shown in Figure 10.

The calculation of the three 2D-ROC curves for the area under the curve (AUC) allowed us to determine the detection probability, background suppression rate, and effectiveness of the detector. The 2D-ROC curves were expanded using the principle of the confusion matrix. The AUC reflects the performance of the detector. Combined with the AUC of the three types, we have evaluation criteria for the six types of detectors; the formulas are expressed as follows:

(13) $$\begin{array}{c} -1\leq AUC_{OD}=AUC_{\left(D,F\right)}+AUC_{\left(D,\tau \right)}-AUC_{\left(F,\tau \right)}\leq 2 \end{array}$$

(14) $$\begin{array}{c} 0\leq AUC_{TD}=AUC_{\left(D,F\right)}-AUC_{\left(D,\tau \right)}\leq 2 \end{array}$$

(15) $$\begin{array}{c} -1\leq AUC_{BS}=AUC_{\left(D,F\right)}-AUC_{\left(F,\tau \right)}\leq 1 \end{array}$$

(16) $$\begin{array}{c} -1\leq AUC_{TD-BS}=AUC_{\left(D,\tau \right)}-AUC_{\left(F,\tau \right)}\leq 1 \end{array}$$

(17) $$\begin{array}{c} 0\leq AUC_{ODP}=AUC_{\left(D,\tau \right)}+\left(1-AUC_{\left(F,\tau \right)}\right)\leq 2 \end{array}$$

(18) $$\begin{array}{c} 0\leq AUC_{SNPR}=\frac{AUC_{\left(D,\tau \right)}}{AUC_{\left(F,\tau \right)}} \end{array}$$

The evaluation criteria from (13) to (18) represent the overall detection (*AUC_OD_*), target detectability (*AUC_TD_*), background suppressibility (*AUC_BS_*), joint target detectability with background suppressibility (*AUC_TD_*_−*BS*_), overall detection probability (*AUC_ODP_*), and signal-to-noise probability ratio (*AUC_SNPR_*). The higher the value of these six criteria, the better the result. In (14), *AUC_TD_* combines *AUC*_(*D*,*F*)_ and *AUC*_(*D*,*τ*)_; the higher its value, the better the target detectability of the detector. In (15), *AUC_BS_* subtracts *AUC*_(*F*,*τ*)_ from *AUC*_(*D*,*F*)_; the higher its value, the better the detector background suppressibility. In (16), *AUC_TD_*_−*BS*_ considers the case where *AUC_TD_* and *AUC_BS_* are different, and it subtracts *AUC*_(*F*,*τ*)_ from *AUC*_(*D*,*τ*)_; the higher its value, the better the detector target background suppression. In (17), *AUC_ODP_* considers target detection and background detectability; the higher its value, the higher the overall detection rate of the detector. *AUC_SS_* represents the true positive (TP) or sensitivity, and *AUC_SF_* represents the true negative (TN) or specificity. The *AUC_SNPR_* of (18) is attributed to the signal-to-noise ratio (SNR) in communication signal processing; the higher its value, the better the detector. The 2D-ROC and 3D-ROC curves for each detector are shown in Figure 10. Table 2 lists the values of the AUC for the 2D-ROC curve; the six evaluation criteria for the detectors are listed in Table 2. Table 3 shows that the DNN is excellent in terms of the target detection rate and effectiveness of the detector. The background suppression abilities of the four detectors are very similar. The evaluation criteria in Table 3 indicate that the DNN is the best detector. However, our detection method is based on pixel detection. When the tumor area of a case is small, fewer pixels are detected, and therefore, the value of the AUC changes significantly.

### 3.4. Quantitative Analysis of Breast Tumors

The quantitative parameters of breast tumors were calculated using the IVIM model. We used ADC, D, D*, PF, and Slope to calculate the quantitative parameters, and the parameter signal decay slope (Slope) was added simultaneously. We provide the quantitative parameters of the detection results for different detectors in Table 4. The quantitative parameters of the tumor detected by our detector were relatively close to those of the ground truth. We converted the slope curve into a signal intensity decay curve plot, and through this curve, we determined whether the detected breast tissue was similar.

In this study, the original classification of tumors as mass and non-mass has been reframed using the terms high cellularity and low cellularity to better reflect the microstructural characteristics of the tumor regions. Mass tumors, with densely packed cellular structures, are described as high cellularity regions, while non-mass tumors, with more dispersed cellular arrangements, correspond to low cellularity regions.

**Figure 11:**
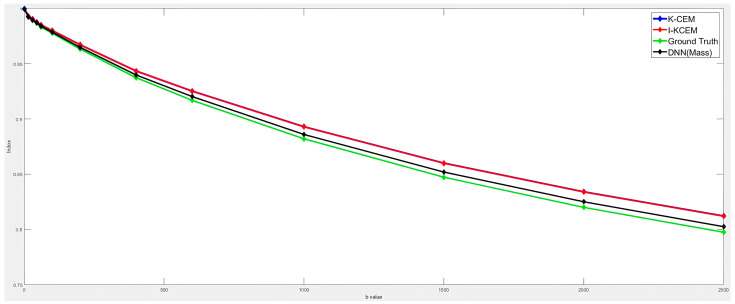
Signal decay slope curves for K-CEM, I-KCEM, DNN, and ground truth with different b-values.

Figure 11 depicts the signal decay slope curves for all detectors, with the similarity in the signal decay slope curves suggesting that the detected results correspond to tumor regions. The DNN is capable of identifying tumors with high and low cellular fractions. The quantitative parameters, detailed in Table 5, illustrate the heterogeneity of the tumors detected by the DNN.

**Figure 12:**
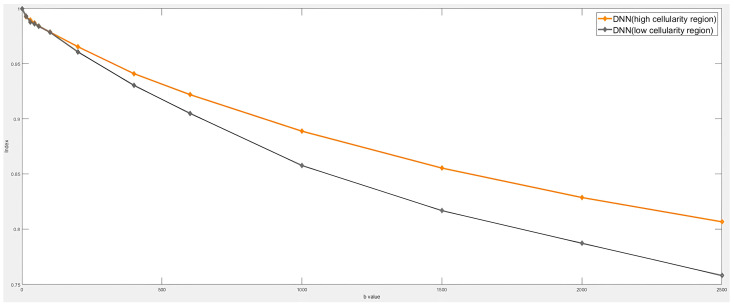
Signal decay slope curves for the DNN in different cellularity with different b-values.

These quantified metrics indicate that water molecules exhibit greater confinement in tumors characterized by high cellular granularity. The signal decay slope curve in Figure 12 also separates the high cellularity portion of the tumor from the low cellularity portion. The signal decay slope curve of the high cell density structure part is on the top, and the low cell density structure part is on the bottom.

The data presented in Table 6 summarizes the mean quantitative parameters of dense tumors in a cohort of 22 patients. The key metrics evaluated include the apparent diffusion coefficient (ADC), the signal decay slope, and the diffusion parameters, such as D*, D, and perfusion fraction (PF). For mass tumors, the mean ADC value is reported as 1.19 × 10⁻^3^ mm^2^/s, indicating restricted water diffusion within dense tumor tissues. The signal decay slope is −0.18 × 10⁻^3^, reflecting the rate of signal intensity reduction. The mean pseudo-diffusion coefficient (D*) is 6.24 × 10⁻^3^ mm^2^/s, while the diffusion coefficient (D) is 0.831 × 10⁻^3^ mm^2^/s. The perfusion fraction (PF) accounts for 24.18%, representing the vascular contribution to overall diffusion measurements. These parameters collectively provide insights into the microstructural and functional characteristics of dense tumors in this patient population

Table 7 presents the computational complexity of the DNN when applied to IVIM images, detailing the number of parameters and the training time required. The DNN model involves 26,115 parameters and completes training within 0.30 h.

## 4. Discussion

The primary focus of this research was to evaluate and compare the effectiveness of deep neural networks (DNNs) against kernel-based constrained energy minimization (KCEM) and iterative KCEM (I-KCEM) methods for breast tumor segmentation and characterization using IVIM-DWI and hyperspectral imaging. Specifically, IVIM imaging was employed to extend conventional diffusion-weighted imaging (DWI) by explicitly decomposing the signal decay into components representing true molecular diffusion (D) and capillary microcirculation (pseudo-diffusion or D*). This approach enables a comprehensive, detailed, and non-invasive assessment of breast tumor heterogeneity without the potential health risks associated with gadolinium-based contrast agents. Our findings highlight that combining IVIM-DWI with hyperspectral analysis and deep learning algorithms offers significant potential for advancing breast tumor characterization and clinical diagnostics.

Although our dataset consisted exclusively of patients diagnosed with mass-type breast cancer, the clinical utility of this research is significant due to its advancement of non-invasive diagnostic approaches and its detailed characterization of breast tumors. First, this method eliminates the dependency on gadolinium-based contrast agents traditionally employed in DCE-MRI, thereby offering a safer alternative, particularly crucial for patients at risk of gadolinium retention or nephrogenic systemic fibrosis. Second, by characterizing breast tumor heterogeneity using quantitative diffusion parameters (ADC, D, D*, and PF) and signal decay curves, our method provides critical insights into tumor microstructure and functional properties. Notably, the DNN model demonstrated the capability to differentiate between high- and low-cellularity regions, potentially serving as an important biomarker for assessing tumor aggressiveness and guiding personalized treatment strategies. Therefore, while the Dice similarity coefficient was specifically employed to evaluate the segmentation accuracy of existing tumor regions rather than binary cancer detection, our overall approach represents a robust, safe, and effective tool with substantial clinical implications for breast cancer diagnosis and management.

### 4.1. Task Specificity

The research aims to classify hyperspectral vectors of individual pixels within breast tissues as either tumor or normal tissue. This task does not necessarily require highly specialized architecture like convolutional neural networks (CNNs), which excel at image recognition tasks involving object shapes and edge detection. The DNN multilayer perceptron model was selected because it is particularly suited to IVIM data analysis, as each pixel’s diffusion-weighted signals across multiple b-values form distinct vectors. Even with fewer IVIM images, sufficient pixel-level data are available for robust analysis. The fully connected DNN, due to its simpler architecture, effectively classifies pixel-level hyperspectral data.

### 4.2. Computational Efficiency

More complex neural networks typically have a higher number of parameters, requiring more computational resources and longer training times. The sources report that the chosen DNN model has 26,115 parameters and completes training within 0.30 h. Using a simpler DNN architecture likely contributes to the following computational efficiency:

1. Interpretability and Explainability

Simpler models are generally easier to interpret and understand. The sources emphasize the importance of analyzing quantitative parameters (ADC, D, D*, and PF) and signal decay curves for understanding tumor characteristics. A simpler DNN model might facilitate the interpretation of its output in relation to these quantitative parameters, aiding in clinical decision-making.

2. Focus on Hyperspectral Analysis

The research primarily emphasizes the application of hyperspectral imaging techniques to IVIM-DWI data. The use of a DNN is presented as a tool to effectively analyze the hyperspectral vectors, and the comparison is focused on contrasting the DNN with traditional hyperspectral analysis methods (KCEM and I-KCEM). Exploring more complex neural network architectures might have shifted the focus away from the core hyperspectral analysis aspect.

### 4.3. Considerations for Future Research

While the chosen DNN demonstrates good performance, exploring more complex neural network models in future research could provide the following benefits:

1. CNNs for Feature Extraction

CNNs excel at automatically learning relevant features from image data, potentially improving detection accuracy and reducing the need for manual feature engineering.

2. Recurrent Neural Networks (RNNs) for Temporal Analysis

The sources do not explicitly mention the temporal aspect of IVIM-DWI data acquisition. RNNs, designed to handle sequential data, could be explored to leverage any temporal information within the imaging sequences.

3. Hybrid Architectures

Combining different neural network types, such as CNNs and RNNs, could potentially lead to even more robust and accurate tumor detection models.

Further research and experimentation would be needed to determine the optimal neural network architecture for this specific task.

## 5. Conclusions

In this research, we introduced an innovative analytical method utilizing IVIM-DWI, which extends traditional DWI by separating signal decay into components of pure molecular diffusion (D) and capillary microcirculation (pseudo-diffusion or D*). This allows for comprehensive characterization of breast tumor tissues without relying on contrast agents, making it a safer and non-invasive alternative for clinical diagnosis. This method incorporates hyperspectral technology into MR imaging to detect breast tumors. By analyzing these tumors with quantitative parameters, we generated hyperspectral image stacks from IVIM-DW images with diverse b-values and utilized hyperspectral imaging technology for image processing. Tumor detection involved vectorizing IVIM-DW imaging features and processing them through a deep neural network (DNN) for classification.

We also investigated kernel-based constrained energy minimization (KCEM) and iterative KCEM methods, which were found to be less effective in identifying breast tissue heterogeneity compared to the DNN. The DNN model demonstrated superior performance in detecting variations in cell density within tumors, achieving a Dice similarity coefficient of approximately 87% and a Jaccard index close to 76%. Specifically, our model differentiates solid tumors from pseudo lipomas by exploiting distinct IVIM characteristics: solid tumors typically exhibit restricted diffusion (lower ADC and D values), while pseudo lipomas generally demonstrate higher ADC values, indicating freer diffusion. Furthermore, using a 3D-ROC method to assess detector performance, the DNN consistently showed the highest effectiveness.

The quantitative analysis confirmed that tumors exhibit distinct diffusion characteristics, reflected in parameters such as the apparent diffusion coefficient (ADC), pure diffusion and pseudo-diffusion coefficients (D and D*), perfusion fraction (PF), and signal decay slopes. These metrics, along with the signal decay slope curves, provide insights into the microstructural and functional traits of breast tumors, emphasizing the ability of the DNN to differentiate high- and low-cellularity tumor regions with high reliability. We also highlight the importance of interpretability and explainability in the chosen DNN model. Connecting the DNN output to interpretable parameters like signal decay curves could enhance clinical decision-making, and therefore, it can be inferred that the signal attenuation curve has the potential to be used as a biomarker for breast tumor detection.

This extensive study, which analyzed a wide array of cases and quantitative tumor parameters, underscores the feasibility of hyperspectral imaging and deep learning as robust diagnostic tools. It serves as a valuable resource for clinical diagnosis and the advancement of breast cancer detection methods.

The analysis of quantitative parameters, including signal decay curves, helps to understand tumor characteristics. This suggests that signal decay curves contain valuable information related to tumor properties.

Future work will focus on expanding the dataset to include diverse tumor types, exploring more advanced neural network architectures, and further improving tumor detection efficiency and accuracy using IVIM-DW images.

## Figures and Tables

**Table 1:** Dice similarity coefficient and Jaccard index of each method.

%	K-CEM	I-KCEM	DNN
Dice	78.49	78.55	**86.56**
Jaccard	64.60	61.37	**76.30**

**Table 2:** Six evaluation criteria of the detectors.

Detector	*AUC_TD_*	*AUC_BS_*	*AUC_TD−BS_*	*AUC_ODP_*	*AUC_OD_*	*AUC_SNPR_*
K-CEM	1.378	0.928	0.388	1.388	1.347	13.7
Iterative K-CEM	1.374	0.906	0.378	1.378	1.329	9.346
DNN	1.523	0.946	0.505	1.505	1.487	14.97

**Table 3:** AUC of the three 2D-ROC curves for three detectors.

Detector	*AUC_(D,F)_*	*AUC_(D,τ)_*	*AUC_(F,τ)_*
K-CEM	0.959	0.419	0.033
Iterative K-CEM	0.951	0.423	0.047
DNN	0.982	0.541	0.036

**Table 4:** The quantitative analysis of the ground truth, K-CEM, I-KCEM, and DNN.

Detector	*ADC* (10^−3^ mm^2^/s)	Signal Decay Slope (10^−3^)	D* (10^−3^ mm^2^/s)	*D* (10^−3^ mm^2^/s)	*PF* (%)
Ground Truth	1.16	−0.169	6.27	0.764	25.12
K-CEM	1.08	−0.157	6.50	0.733	22.85
Iterative K-CEM	1.08	−0.157	6.49	0.733	22.85
DNN	1.12	−0.165	6.31	0.751	23.99

**Table 5:** Quantitative parameters of the DNN in different cellularity.

Different Cellularity	*ADC* (10^−3^ mm^2^/s)	Signal Decay Slope (10^−3^)	D* (10^−3^ mm^2^/s)	*D* (10^−3^ mm^2^/s)	*PF* (%)
DNN (High Cellularity)	1.10	−0.161	6.35	0.739	23.25
DNN (Low Cellularity)	1.31	−0.196	7.32	0.872	27.93

**Table 6:** Mean of the quantitative parameters of dense tumors in 22 patients.

Detector	*ADC* (10^−3^ mm^2^/s)	Signal Decay Slope (10^−3^)	D* (10^−3^ mm^2^/s)	*D* (10^−3^ mm^2^/s)	*PF* (%)
Mass Tumor	1.19	−0.18	6.24	0.831	24.18

**Table 7:** Computational complexity of the DNN using IVIM images.

METHOD	Parameters	Time (h)
DNN	26,115	0.30

## Data Availability

The datasets analyzed during the current study are not publicly available due to restrictions imposed by the Institutional Review Board (IRB) and the Human Subjects Research Ethics Committee of Taichung Veterans General Hospital. These data contain sensitive patient information and are protected to ensure patient confidentiality. Requests for access to the data may be considered on a case-by-case basis and require appropriate ethical approvals.
